# Developmental changes of poly(ADP-ribose) polymerase expression in *Sarcophaga peregrina*

**Published:** 2004-07-01

**Authors:** Mitsuko Masutani, Miyoko Ikejima, Sofia Mariotto, Tadashige Nozaki, Shoichiro Kurata, Shunji Natori, Hiroyasu Esumi, M. J. A Takashi Sugimura

**Affiliations:** *) Biochemistry Division, National Cancer Center Research Institute, 5-1-1, Tsukiji, Chuo-ku, Tokyo 104-0045, Japan; **) Faculty of Pharmaceutical Sciences, University of Tokyo, 7-3-1, Hongo, Bunkyo-ku, Tokyo 103-0033, Japan

**Keywords:** Poly(ADP-ribose) polymerase (PARP), *Sarcophaga*, development, expression, PARP activity

## Abstract

To elucidate the physiological role of poly(ADP-ribose) polymerase (PARP), we studied the levels of *PARP* mRNA and protein during the developmental stages of *Sarcophaga peregrina*. *PARP* mRNA expression changed remarkably throughout the developmental stages. The level of *PARP* mRNA (the molecular ratio of *PARP* mRNA to the total RNA) was highest in unfertilized eggs and that of PARP protein (the molecular ratio of PARP protein to the total protein of the crude extract) was high in unfertilized and fertilized eggs and in 1st instar larvae. During the embryogenesis period, the levels of *PARP* mRNA and protein gradually decreased. The levels of *PARP* mRNA during larval and pupal periods became less than about 5% of that in unfertilized eggs. After the emergence of adult flies, the levels of *PARP* mRNA and protein increased both in female and male flies. PARP activity normalized with the total amount of protein in the crude extract changed in parallel to the level of PARP protein throughout the developmental stages. The biological significance of the drastic change of mRNA and protein levels of *PARP* still remains to be clarified.

## Introduction

Poly- and monoADP-ribosylation reactions are involved in various physiological processes in animals, including vertebrates and invertebrates. PolyADP-ribosylation is catalyzed by poly(ADP-ribose) polymerase-1 (PARP-1) and other PARP family members recently discovered in vertebrates.1) MonoADP-ribosyl transferases in vertebrates were found as glycosylphosphatidylinositol-anchored, secreted or intracellular proteins and are possibly involved in the regulation of signal transductions.2) Pierisin isolated from the cabbage butterfly (*Pieris rapae*) monoADP-ribosylates guanine residues of DNA3) and causes apoptosis.4) The expression of pierisin is specifically induced during the late stage of larvae and early stage of pupae.4) The *in vivo* evidence for roles of poly- and monoADP-ribosylation reactions in development, differentiation and pathogenesis, including cancer formation, has been accumulating.

Poly(ADP-ribose) polymerase-1 (PARP-1) is present in nuclei and centrosomes5) and polyADP-ribosylates proteins upon activation by DNA strand breaks.6),7) The involvement of PARP in DNA damage recognition, DNA repair,8),9) DNA replication,10) cell death11),12) and differentiation,13),14) as well as cancer development15)–17) has been reported. Biological and biochemical studies, including those using *PARP* knockout mice and *Drosophila* are available.

*Parp-1* disrupted *Drosophila* showed a decrease of expression in the genes located in puff loci, accompanied by the lack of puff formation.18) Transgenic flies that overexpress *PARP* showed aberrant organization of F-actin and tissue polarity.19) The presence of PARP family proteins has been recently characterized in vertebrates, but the presence of PARP family proteins other than PARP-1 orthologs has not been described for invertebrates.

We previously reported the presence of PARP activity in the fleshfly, *Sarcophaga peregrina*, and purified PARP20) and cloned *Sarcophaga PARP* cDNA.21) *Sarcophaga* has been used as a good experimental system for development and morphogenesis studies.22)–24) Here, we examined changes in the levels of *PARP* mRNA and protein at different stages during the development of *Sarcophaga* and found that the levels of *PARP* mRNA and protein were strictly regulated during development.

## Materials and methods

### Insects

The fleshfly *Sarcophaga peregrina* was kept at 27 °C. Third instar larvae were maintained under dry conditions to ensure synchronous pupation. Adult flies, which emerged 10 days after pupation, were cultured at 25 °C with skimmed milk and water. For the preparation of unfertilized eggs, female flies were separated from male flies soon after emergence, reared separately, and unfertilized eggs were harvested from 5-day-old females. Fertilized eggs and embryos were collected from females after start of mating using 2-day-old females as described by Takahashi *et al*.25) An embryonic cell line, *Sarcophaga* NIH-Sape-4 cell was cultured as described.26)

### Northern blot analysis

Total RNA was extracted by the method of Chomczynski and Sacchi, with minor modifications.21) Unfertilized and fertilized eggs, larvae, pupae, and adult flies were homogenized with 5 volumes of extraction buffer (4 M guanidinium thiocyanate, 25 mM sodium citrate (pH 7.0), 0.1 M *β*-mercaptoethanol, 0.5% sodium lauryl sarcosinate). After extraction with acid-phenol and chloroform, RNA was precipitated twice with isopropanol and redissolved in water. The level of *PARP* mRNA (the molecular ratio of *PARP* mRNA to the total RNA) was measured by northern blotting using a 0.3 kb ^32^P-labeled *Sarcophaga* PARP cDNA probe, corresponding to codons 721 to 818.21) Ten or 20 μg of total RNA was used for agarose gel electrophoresis and the ethidium bromide staining was carried out to confirm the equal loading of RNA. The probe was prepared with a Multi-Prime Labelling Kit (Amersham, Buckinghamshire, United Kingdom). Hybridization was done at 37 °C for about 12 hours in a solution containing 50% formamide as previously described.21)

### Preparation of Sarcophaga crude cell extract

Frozen materials were homogenized in an extraction buffer containing 50 mM Tris-HCl (pH 7.5), 17% glycerol, 1 mM dithiothreitol, 50 mM NaHSO_3_, 0.5 mM phenylmethylsulfonyl fluoride and 5 μg/ml each of soybean trypsin inhibitor, leupeptin, antipain, pepstatin and chymostatin. The extracts were centrifuged at 128,000 × *g* for 30 min at 4 °C in a Beckman TLA100.2 rotor, and supernatants were stored as crude extracts at −80 °C. Protein quantification was carried out using a Protein Assay Kit (Bio-rad, Hercules, CA, USA).

### PARP activity measurement

Samples were incubated for 10 min at 25 °C in the assay buffer (0.1 ml) consisting of 50 mM Tris-HCl (pH 8.0), 10 mM magnesium acetate, 1 mM dithiothreitol, 100 μM ^32^P-NAD (8 Ci/mmole), 20 μg/ml activated DNA (Sigma, St. Louis, MO, USA), and 20 μg/ml calf thymus histone type IIA (Sigma). The reaction was stopped by the addition of 10% trichloroacetic acid, after which acid-insoluble radioactivity was counted.

### Western blot analysis

Rabbit polyclonal antiserum against purified *Sarcophaga* PARP21) was used in a dilution of 1 : 200. ^125^I-Anti-rabbit IgG (Amersham), or ^35^S-protein A (Amersham) at 0.1 μCi/ml was used as a second antibody. The level of PARP protein was measured as the molecular ratio of PARP protein to the total protein of the crude extract).

## Results

### Expression of PARP during developmental stages

**Fig. 1:**
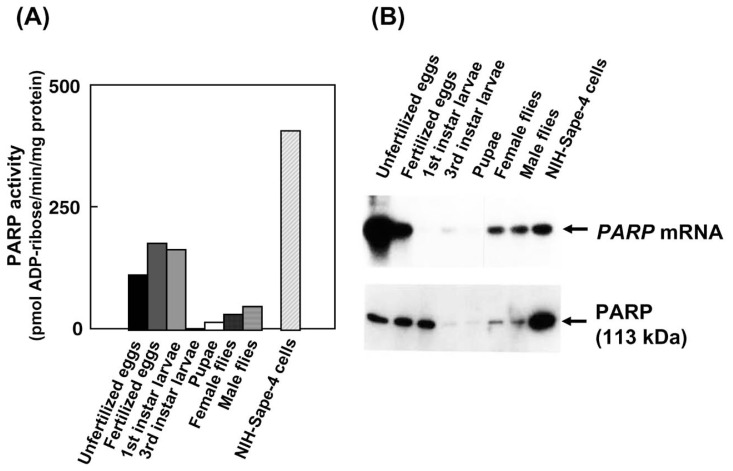
The levels of PARP activity and mRNA in developmental stages of *Sarcophaga*. Unfertilized eggs obtained from 5-day-old females, fertilized eggs obtained from 6-day-old females (corresponding to day 0 embryos), and pupae (8 days after pupation) were used for preparation of total RNA and proteins as described in Materials and methods. Unfertilized and fertilized egg consist of an oocyte and zygote, respectively, and also contains a layer of follicle cells, and nurse cells. (A) Activity of *Sarcophaga* PARP in each developmental stage. Bars represent means of duplicate or triplicate measurements. (B) Northern and western blot analysis of PARP. In northern blot analysis, 10 μg of total RNA prepared from *Sarcophaga* bodies at various stages of development was used. Ethidium bromide staining of the agarose gel confirmed the equal loading of total RNA. 0.3 kb *Sarcophaga* PARP cDNA was used as a probe. In western blot analysis, a crude extract of 30 μg protein obtained from *Sarcophaga* of each developmental stage was used. PARP was detected with anti-*Sarcophaga* PARP polyclonal antibody. ^125^I-Anti-rabbit IgG was used as a second antibody. The arrow indicates 113 kDa PARP.

As shown in Fig. 1(A), PARP activity normalized with the total amount of protein in the crude extract was high in unfertilized eggs (obtained from 5-day-old females), in fertilized eggs (obtained from mated 6-day-old females, day 0 embryo), and in 1st instar larvae. However, it was low in 3rd instar larvae and in the pupae (8 days after pupation). PARP activities in both adult female and male flies were about 25% of that in unfertilized eggs. Western blot analysis of PARP protein, shown in Fig. 1 (B), corresponded well with the activity change. The mRNA levels were highest in unfertilized egg and decreased approximately to half in fertilized eggs (day 0 embryo). Our preparation of unfertilized and fertilized eggs mainly consisted of oocytes and zygotes, respectively, and also contained a layer of follicle cells, which are derived from somatic cells and surround the surface of oocytes, and nurse cells, which are derived from germ cells and show high ploidy as well. We could not exclude the possibility that the preparation of fertilized eggs contain a small percentage of unfertilized eggs. An unfertilized egg was estimated to contain approximately 2 × 10^6^ *PARP* transcripts. In contrast, *PARP* mRNA level was unexpectedly low in 1st instar larvae and 1st instar larvae are estimated to contain less than 3% of that in unfertilized eggs. Third instar larvae had a two-fold higher level of *PARP* mRNA than that of 1st instar larvae. In day 8 pupae, the level of *PARP* mRNA was lower than 10% of that in unfertilized eggs. It is also noted that PARP activity was higher in pupae (8 days after pupation) than in 3rd instar larvae. After emergence of adult female and male flies, *PARP* mRNA levels rapidly increased to 50% of that in unfertilized eggs. Thus the levels of *PARP* mRNA and protein changed markedly throughout the developmental stages. The *PARP* mRNA and PARP protein levels correlated well during each stage except for 1st instar larvae.

### Expression of PARP in embryogenesis

**Fig. 2:**
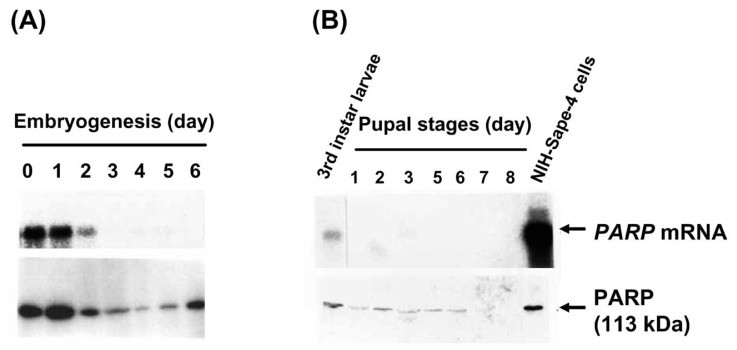
Changes in the levels of *PARP* mRNA and protein during embryogenesis and pupal stages. (A) Changes during embryogenesis. The upper panel shows northern blot analysis using 20 μg of the total RNA prepared from *Sarcophaga* embryos. Ethidium bromide staining of the agarose gel confirmed the equal loading of total RNA. A 0.3 kb *Sarcophaga* PARP cDNA probe was used. The lower panel shows western blot analysis of crude extract of 30 μg protein from *Sarcophaga* eggs. PARP was detected with anti-*Sarcophaga* PARP polyclonal antibody. ^125^I-anti-rabbit IgG was used as a second antibody. The arrow indicates 113 kDa PARP. (B) Changes during pupal stages. The upper panel shows northern blot analysis using 20 μg of the total RNA prepared. 0.3 kb *Sarcophaga PARP* cDNA probe was used. The lower panel is the western blot analysis of a crude extract of 30 μg protein. PARP was detected with anti-*Sarcophaga* PARP polyclonal antibody. ^35^S-protein A was used as a second antibody. The arrow indicates 113 kDa PARP.

*Sarcophaga* is ovoviviparous, with embryonic development usually starting in the ovary of 6-day-old female flies, and 1st instar larvae being laid 5 days later. In order to ensure the simultaneous embryogenesis of the flies, pupation stages and the period of emergence of adult flies were synchronized. We collected embryos from the ovary of female flies every day from day 6 to day 11 after emergence (corresponding to days 0 to 6 of embryogenesis). Northern and western blot analyses for *PARP* expression are shown in Fig. 2 (A). In northern blot analysis, *PARP* mRNA levels decreased gradually to an almost undetectable level during the embryogenesis period (less than 10% of that in day 0 embryo). However, in western blot analysis, the some levels of PARP protein was maintained; it was seen that slight decreases occurred during the early embryonic stages, followed by a small increase in the late embryonic stages. In the late stage of embryogenesis, the storage protein, vitellogenin, which composed more than 90% of the total egg protein, degrades rapidly.27) This vitellogenin degradation is probably related to the apparent increase in the level of PARP protein during the late stages of embryogenesis. As shown here, the embryogenesis period, during which the extensive cell proliferation occurs, was associated with the high level of PARP protein.

### Expression of PARP during pupal stages

As shown in Fig. 2(B), upper panel, lower levels of *PARP* mRNA were observed during the pupation period of 9 days when compared to those of the 3rd instar larvae. However, on the 3rd day of pupation, a small increase in the level of *PARP* mRNA was observed. The PARP protein was present during the first half of the pupal stages but was gradually lost in the latter half (Fig. 2(B), lower panel).

## Discussion

In this paper, we showed that the levels of *PARP* mRNA and protein undergo remarkable changes during the developmental stages of *Sarcophaga peregrina*. *PARP* expression levels at both mRNA and protein were characteristically high in the embryogenesis period but low in the pupal period. Because *PARP* mRNA and protein levels were mostly well-correlated, it is suggested that the level of PARP is transcriptionally regulated. A high level of *PARP* mRNA in the unfertilized eggs was observed and this would suggest the possibility of the maternal storage of *PARP* mRNA. During embryogenesis, extensive cell-proliferation occurs and PARP may play an important role for maintaining the chromatin structure.

In *Drosophila*, a splicing variant of *PARP* mRNA, lacking auto-modification domain, was reported as a minor component during late phase of embryogenesis.28) We could not detect any splicing variants in the northern blot analysis using the cDNA probe encompassing codons 721–818, including those lacking auto-modification domain (data not shown).

During the late stages of embryogenesis and pupal stages, the presence of PARP protein was observed, even though the *PARP* mRNA content was almost undetectable. Although we could not exclude the possibility of the presence of splicing variants undetectable by the probe encompassing codons 721–818, which we used for northern blot analyses, there is a possibility of some regulations at the post-transcriptional level, either by the efficient translation of *PARP* mRNA or the stabilization of PARP protein.

During the larval stage, the level of *PARP* mRNA was relatively low, although in 3rd instar larvae, a small increase in the level of *PARP* mRNA was observed. At the pupal stage, the level of *PARP* mRNA was very low. However, the PARP protein was present during the first half of pupal stages. In this period, the reconstitution of tissues, apoptosis, and cell proliferation in the imaginal disk occurs. Thus, PARP may be possibly required for these processes. Since the cleavage of mammalian PARP-1 by caspases during apoptosis has previously been reported,29) the possibility of the regulation of PARP level by proteolytic cleavage is present.

There were high levels of both *PARP* mRNA and protein in adult female and male flies, although in the late-stages of pupae, the levels of *PARP* mRNA and protein were almost undetectable. Thus, after the emergence of adult flies, probably during oogenesis and spermatogenesis, PARP expression is likely to be greatly induced at the transcriptional level. In *Drosophila*, it is known that DNA polymerase *α* and proliferating cell nuclear antigen, both of which are responsible for chromosome replication, are strongly expressed in adult females but are only weakly expressed in adult male flies.30) It is interesting that male and female adult *Sarcophaga* showed the same significant levels of *PARP* mRNA and protein.

In conclusion, the levels of *PARP* mRNA and protein are strictly regulated during development of *Sarcophaga*. *Sarcophaga* might be a useful animal model for studies of poly(ADP-ribose) metabolism during development.

## Abbreviation

PARP: poly(ADP-ribose) polymerase
Enzyme: Poly(ADP-ribose) polymerase (EC 2.4.2.30)
